# Fractal-geometry analysis of pediatric posterior fossa tumors – a preoperative tool for prediction of histopathology

**DOI:** 10.1007/s10143-025-04078-9

**Published:** 2026-01-22

**Authors:** Tamás Mezei, János Báskay, Balázs Markia, Péter Várallyay, Péter Banczerowski, Péter Pollner

**Affiliations:** 1 Department of Neurosurgery, Semmelweis University Hungary, Amerikai út 57, 1145 Budapest, Hungary; 2 Department of Neurosurgery and Neurointervention, Semmelweis University Hungary, Amerikai út 57, 1145 Budapest, Hungary; 3 Data-Driven Health Division of National Laboratory for Health Security, Health Services Management Training Centre, Semmelweis University Hungary, Kútvölgyi út 2, 1125 Budapest, Hungary; 4 Department of Biological Physics, Eötvös Loránd University Hungary, Pázmány Péter sétány 1/a, 1117 Budapest, Hungary

**Keywords:** Fractal geometry analysis, Radiomics, Pediatric posterior fossa tumor, Histology, Prediction

## Abstract

The most common types of pediatric posterior fossa tumors are pilocytic astrocytomas, embryonal tumors, and ependymomas. Preoperative knowledge of the pathological diagnosis may facilitate optimal surgical management. The complex geometry of tumors can be characterized by using different metrics, such as fractal geometry parameters that play an important role in the description of irregular, rough shapes. Our aim was to perform a fractal analysis of pediatric posterior fossa tumors and identify potential new radiological biomarkers. We conducted a retrospective clinical study using preoperative MRI images and clinical data from pediatric patients who had undergone surgery for posterior fossa tumors. T1, ceT1, T2 and FLAIR sequences were acquired for all patients. The tumors were segmented using ITK-SNAP software, then fractal analysis, t-tests, Fischer exact tests, logistic regression, and ROC analysis were performed. Forty-four patients met the selection criteria. T-tests evaluating tumor volume, fractal dimension (FD), FLAIR lacunarity index (LI), and the Fischer test assessing the cystic component revealed significant differences among the three main tumor types. The prediction formula was constructed by applying logistic regression to weight the selected factors, achieving an AUC of 0.793 (95% CI = 0.628–0.921). Our study suggests that fractal metrics are useful tools for the preoperative estimation of histological diagnosis.

**Supplementary Information:**

The online version contains supplementary material available at 10.1007/s10143-025-04078-9.

## Introduction

The most prevalent pediatric solid tumors, which primarily affect the central nervous system (CNS), are the leading cause of cancer-related mortality among children under 14 years of age ^[1]^. Unlike in adults, where the supratentorial occurrence is more frequent, 45–60% are located in the posterior fossa in pediatric cases. In terms of histological distribution, the most common are pilocytic astrocytomas (15.3%), embryonal tumors (9.5% in total, of which 64.7% are medulloblastomas, 16.6% atypical teratoid/rhabdoid tumors [AT/RT]) and ependymomas [1]. Clinicopathologically, they are highly heterogeneous, with significant differences in treatment and prognosis.

MRI scans are essential to assess preoperative status, plan surgery and evaluate complications (e.g., hydrocephalus) [2, 3]. An important aspect of preoperative image interpretation is the differentiation of the three main tumor types. For pilocytic astrocytomas and ependymomas, radical resection is necessary to achieve the best possible PFS and OS [4, 5], whereas for medulloblastoma, a residuum of less than 1.5cm^3^ is not associated with a significant reduction in survival [6]. Conventional MRI can identify certain features, but its specificity and sensitivity are not sufficient for preoperative histological diagnosis.

The human body, organs, and tissues have complex geometric structural properties, including abnormal tissues and tumors. Their characterization using only the metrics of classical Euclidean geometry is cumbersome and results in inaccuracies. The complex geometry of tumors can be characterized by their shape properties using different measures. Fractal dimension, introduced by Benoit Mandelbrot [7, 8] in 1977 and 1982, plays an important role in the characterization of irregular, rough shapes. Fractal dimension is a fractional number that provides information about the structural complexity of the shape of an object. Lacunarity index is a measure that describes the amount and spatial distribution of material within a shape or volume, indicating how homogenous or gapped it is. These parameters could be compared between groups of patients and, even more, between unhealthy and healthy controls [9].

The irregular shape and rough surface of tumors may indicate invasive behavior, a malignant histological finding [10]. For brain tumors, the correlation between fractal analysis and WHO classification has been reported in the literature for meningiomas [11, 12], the differentiation of glioblastoma from primary CNS lymphoma (PCNSL) [13], or the survival prediction of patients with glioblastoma based on the fractal parameters of necrosis [14] .

The aim of our research is to identify a novel radiological biomarker by fractal geometry analysis that may improve the estimation of preoperative histological diagnosis to achieve personalized treatment in patients with pediatric posterior fossa tumor.

## Methods

### Patient selection

A single-center retrospective clinical study of the clinical, radiological and histopathological characteristics of pediatric patients undergoing surgery for posterior fossa tumors at our Clinic was conducted. The study was approved by the Local Ethics Committee (IKEB Registration Number: 4/2023) and by the Scientific and Research Ethics Committee of the Hungarian Medical Research Council (ETT-TUKEB Registration Number: BM/26596-1/2023).

We collected data from March 2018 to May 2023. The main inclusion criterion was the availability of the complete DICOM sequence of the preoperative MRI scan (T1, T1c, T2, and FLAIR sequences were mandatory).

The following pre-, intra-, and postoperative clinical parameters were collected:

- Age and sex.
- Presenting symptoms (e.g., hydrocephalus, ataxia, and gaze palsy, etc.)
- MRI findings: localization (cerebellar hemisphere, vermis, pontocerebellar, and fourth ventricle), presence of a cystic component, radiological hydrocephalus, contrast enhancement, and tumor volume.
- Type of surgical resection (e.g., total, subtotal, and biopsy).
- Neuropathological (2021 CNS WHO) diagnosis and features.
- Presence of residuum on the postoperative MRI.
- Presence of spinal metastasis on the pre- or postoperative neuraxis MRI.

### Preprocessing of the MRI sequences

MRI sequences were obtained from multiple institutions and prepared by various scanners, so that the resolution of the collected dataset varied from 0.3 mm^3^ up to 1 mm^3^ per voxel. The sequences were transformed to a uniform resolution of 1 mm^3^ and then coregistered to the ceT1 sequence using the Cancer Imaging Phenomics Toolkit’s BraTS Pipeline [15, 16].

**Fig. 1:**
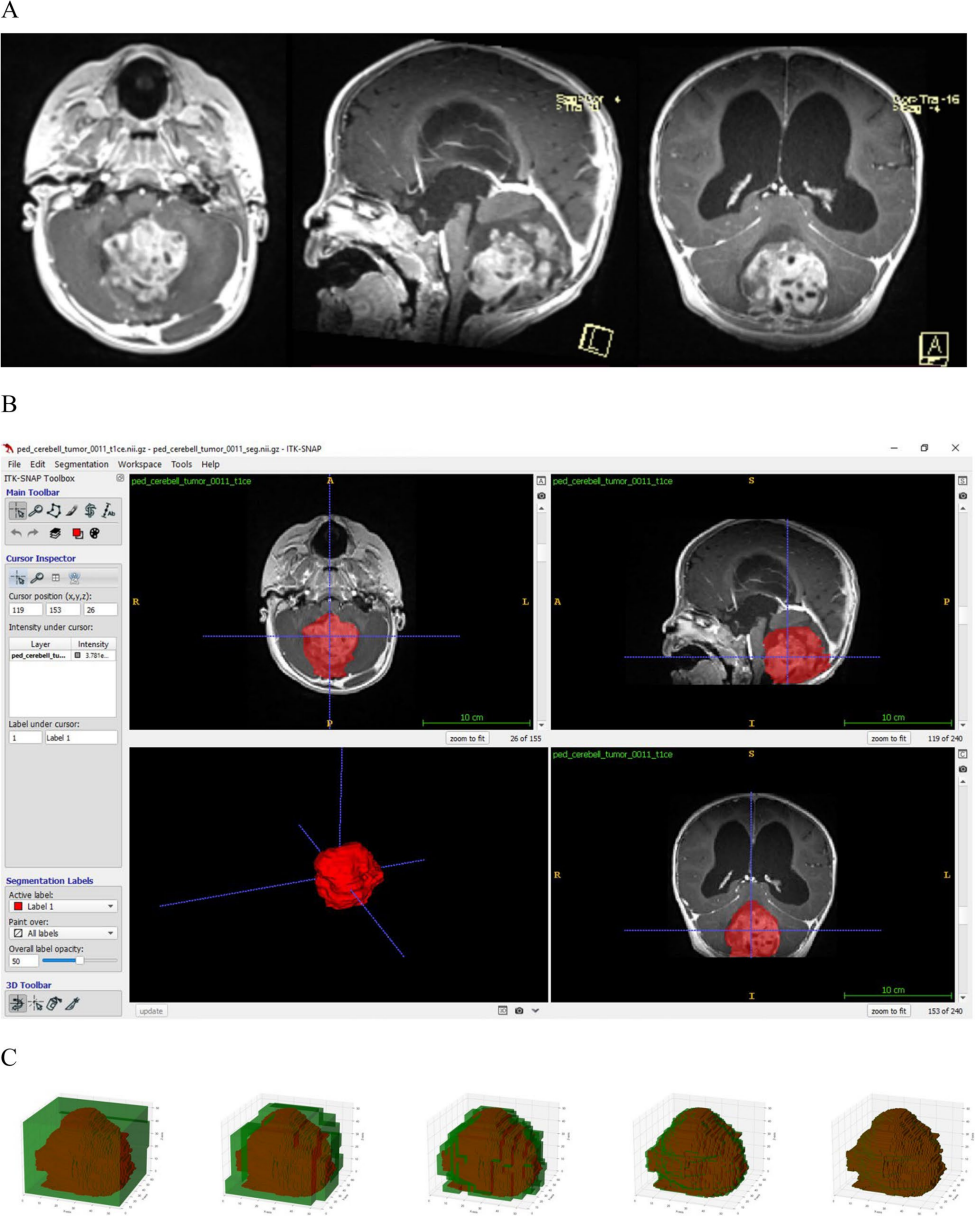
The process of the segmentation and the fractal analysis. **A** T1-weighted contrast-enhanced axial, sagittal, and coronal (left to right) MR images of a pediatric patient with posterior fossa tumor. **B** We used ITK-SNAP for the segmentation of the tumors. We annotated the axial view using polygon mode for every 3–5 slices, and then interpolated the annotations to the rest of the image using the software. Finally, the program created the spatial model of the tumor on which the fractal analyses were run. **C** The flowchart shows the box counting methodology used in the fractal analysis

Abnormal signal areas on ceT1-weighted MRI were manually delineated using ITK-SNAP (version 3.8.0, PICSL, Philadelphia, USA) as tumor regions of interest (ROI) by a neurosurgeon. All ROI were subsequently reviewed by a senior neurosurgeon with 16 years of experience who was blinded to the clinical data of each patient, and then independently reannotated by a neuroradiologist for interobserver agreement (Figs. 1A and B).

### Fractal analysis

The fractal analysis was performed using an in-house Python tool, FracND, which uses a CPU-parallelized sliding window for box counting and lacunarity calculation in N dimensions.

The fractal dimension was obtained from the 3D binary segmentation masks, by calculating the number of non-empty windows (‘box count’) (Fig. 1C) for a given scale and then finding the slope of the log-log plot between the box count and the inverted window size^8,9^.

Lacunarity for a given scale was obtained by calculating the lacunarity coefficient, which is the square of the coefficient of variation for each window, and then calculating the average over all windows. The lacunarity index [17, 18] used to describe the whole volume across scales is the slope of the log-log plot of lacunarity for a given scale and inverted window size.

For various MRI sequences, tumor lacunarity requires the inclusion of voxel values; this was performed in a manner similar to FracLac in ImageJ: voxel values were used as an additional dimension, transforming the 3D voxel volume into a 4D surface, on which FracND could measure lacunarity. For the 4D measurements, subsampling was necessary to speed up calculations, with a subsampling rate of 0.01, meaning that the contents within the sliding window were evaluated randomly at a rate of 1:100, otherwise they were ignored.

### Statistical analysis

Neuropathological diagnoses were compared with clinical categorical variables using Fisher’s exact test and with continuous variables using independent samples t-test, as all continuous variables were found to be normal.

Interobserver agreement for the annotation masks was assessed using Cohen’s kappa index [19]. Agreement between fractal dimension and lacunarity, measured on annotation masks created by neurosurgeons and those made by neuroradiologists, was evaluated using two-way interclass correlation.

Multivariable logistic analyses were performed, first on fractal parameters alone, and then with the introduction of clinical parameters. Continuous variables were scaled between 0 and 1. The variables were selected by a separate greedy exhaustive search for fractal parameters and a separate search for clinical parameters, both targeting maximal AUC obtained from receiver operating characteristic (ROC) curve analysis on the out-of-bootstrap samples and calculating the area under the ROC curve. All selected variables were also confirmed by Fisher’s exact test or independent samples t-test (*p* < 0.05). Overfitting was avoided by bootstrapped sampling with *n* = 100 and replacement (Supplement 1). The scoring system was generated from the mean regression weights across the bootstrapped samples by absorbing the scaling factors of continuous variables in the logits.

Statistical analyses were performed using Python and R: t-tests for independent samples were conducted with SciPy; logistic regression and Cohen’s kappa with Scikit-Learn; interclass correlation with Pingouin, and Fisher’s exact tests with R.

## Results

### Descriptive statistics

Finally, we identified 44 pediatric patients who met the selection criteria, 25 (57%) of them were female and 19 (43%) were male. The median age of the population was 6.5 years. The most common histological features were pilocytic astrocytoma (38.6%) and medulloblastoma (29.5%); the other third of the population was heterogeneous (e.g., ganglioglioma, posterior fossa ependymoma, hemangioblastoma, AT/RT, choroid plexus papilloma, and epidermoid cyst, etc.), so for further statistical evaluation, we treated them together as the category of “other tumor”, and focused on these three categories. Radical tumor resection was performed in 81.8% of the patients. The incidence of extracranial metastasis was 4.5%. The median tumor volume was 36.3cm^3^ (min = 2.8 cm^3^, max = 112.9 cm^3^).

### Comparison of histology with clinical parameters

No significant difference in presenting symptoms was observed between patients with different histologies. Local symptoms (e.g., ataxia, and gaze disturbance) had higher odds of occurrence in the pilocytic astrocytoma group (OR = 2.94 (95% CI = 0.673–15.671)), but the difference was not significant (*p* = 0.12).

In comparison with medulloblastoma and pilocytic astrocytoma, the “other tumors” were significantly less likely to be located in the vermis (*p* = 0.05, OR = 0.20 [95% CI = 0.018–1.126]). A cystic component was significantly common in pilocytic astrocytoma (*p* = 0.02, OR = 3.40 [95% CI = 0.780-18.121]) and less likely in medulloblastoma (OR = 0.19 [95% CI = 0.033–0.893]), but not significant (*p* = 0.11).

**Fig. 2:**
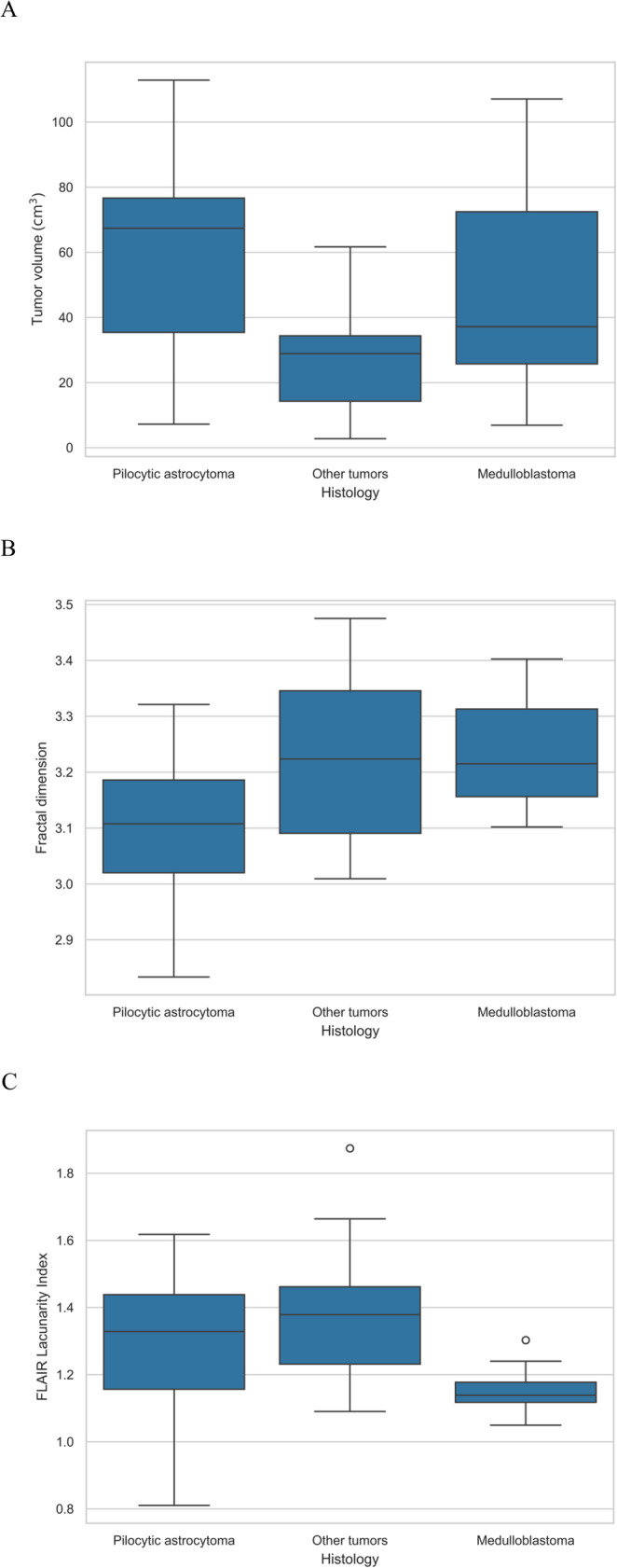
Boxplots comparing tumor volume (**A**), fractal dimension (**B**) and lacunarity index (**C**). The thin outer edges represent the minimum and maximum, the bottom of the wide middle section represents the 25th percentile, the top the 75th percentile, and the middle line represents the group median (50th percentile). Outliers are indicated by white circles

T-tests were used to compare the volumes between the histopathology groups. We found that the “other tumor” subgroup had significantly smaller mean volumes than the medulloblastoma (*p* = 0.04) and pilocytic astrocytoma (*p* = 0.0004) subgroups (Fig. 2A). We attempted to determine if any differences could be identified among the three histological subgroups in terms of the fractal geometry parameters (FD and LI). Pilocytic astrocytoma was well-separated by FD from “other tumor” (*p* = 0.05) and from medulloblastoma (*p* = 0.01) (Fig. 2B). Medulloblastoma was well-separated by FLAIR LI from the other two subgroups (Fig. 2C, *p* = 0.01 for pilocytic astrocytoma and *p* = 0.002 for “other tumor”).

### Interobserver agreement

The interobserver agreement on imaging parameters was excellent: Cohen’s κ = 0.899 (95% CI = 0.809–0.952), intraclass correlation coefficient (ICC) = 0.778 (95% CI = 0.627–0.930).

### Predictive models

Logistic regression was chosen to set up a predictive tool. The metrics used for the evaluation were the results of the ROC analysis, AUC, accuracy, precision, and recall.

The feature set with the highest score is selected. In case of a tie, the preference will be given to the smaller feature sets. During the exhaustive search, fractal parameters are treated independently of all other features. First, we select the optimal fractal parameters, and with this optimal choice, we search exhaustively among the other features. This way we can avoid a combinatorial explosion of the search space.

We observed that bootstrapping yielded more conservative results for accuracy, precision, and recall. However, it significantly reduced the risk of model overfitting compared to cross-validation, especially due to the small sample size of 44.

#### Fractal parameters for the prediction of histopathology

**Fig. 3:**
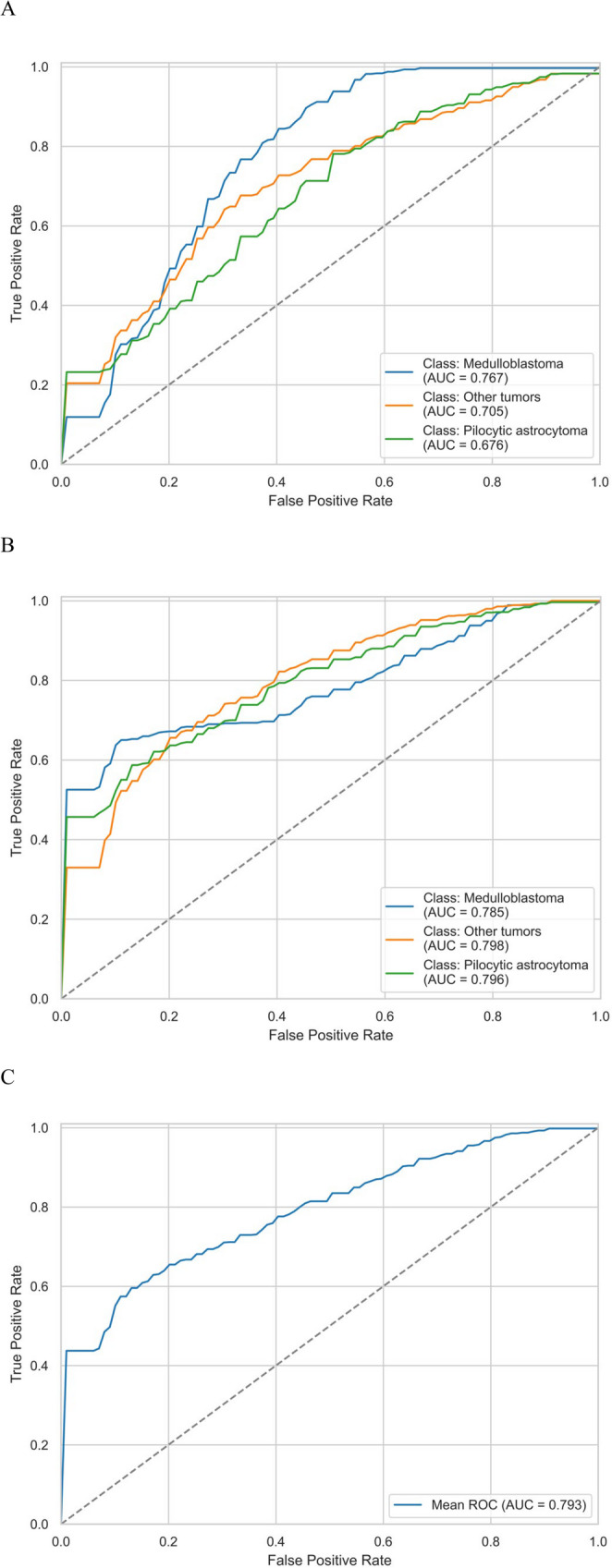
ROC analysis of the prediction model. **A** ROC curves (with CI intervals) and AUC values when only the fractal parameters (fractal dimension and FLAIR lacunarity) were used to predict histopathology. **B** ROC curves (with CI intervals) and AUC values when conventional imaging was added to the fractal parameters to predict histopathology. **C** Mean ROC curve (with CI interval) in Fig. 2B

When only fractal parameters were used for prediction, it was found that the FD and LI were able to separate the three histopathological subgroups. The AUC value was 0.701 for “other tumor”, 0.771 for medulloblastoma, and 0.676 for pilocytic astrocytoma (Fig. 3A) (Supplement 2). The mean AUC was 0.716 (95% CI = 0.607–0.862), accuracy was 0.488 (95% CI = 0.308–0.667), precision was 0.544 (95% CI = 0.251-0.800), and recall was 0.488 (95% CI = 0.308–0.667).

#### Fractal parameters plus clinical features for the prediction of histopathology

The inclusion of conventional imaging parameters substantially improved the predictive capability. When we used FD and LI together with tumor volume and cystic features of the tumors to predict pathological diagnosis, a mean of 0.793 AUC was found (95% CI = 0.628–0.921). (0.798 for “other tumor”, 0.785 for medulloblastoma and 0.796 for pilocytic astrocytoma) (Figs. 3B and C) (Supplement 2), accuracy was 0.616 (95% CI = 0.421–0.812), precision was 0.683 (95% CI = 0.460–0.905), and recall was 0.616 (95% CI = 0.421–0.812).

#### The scoring system

**Fig. 4:**
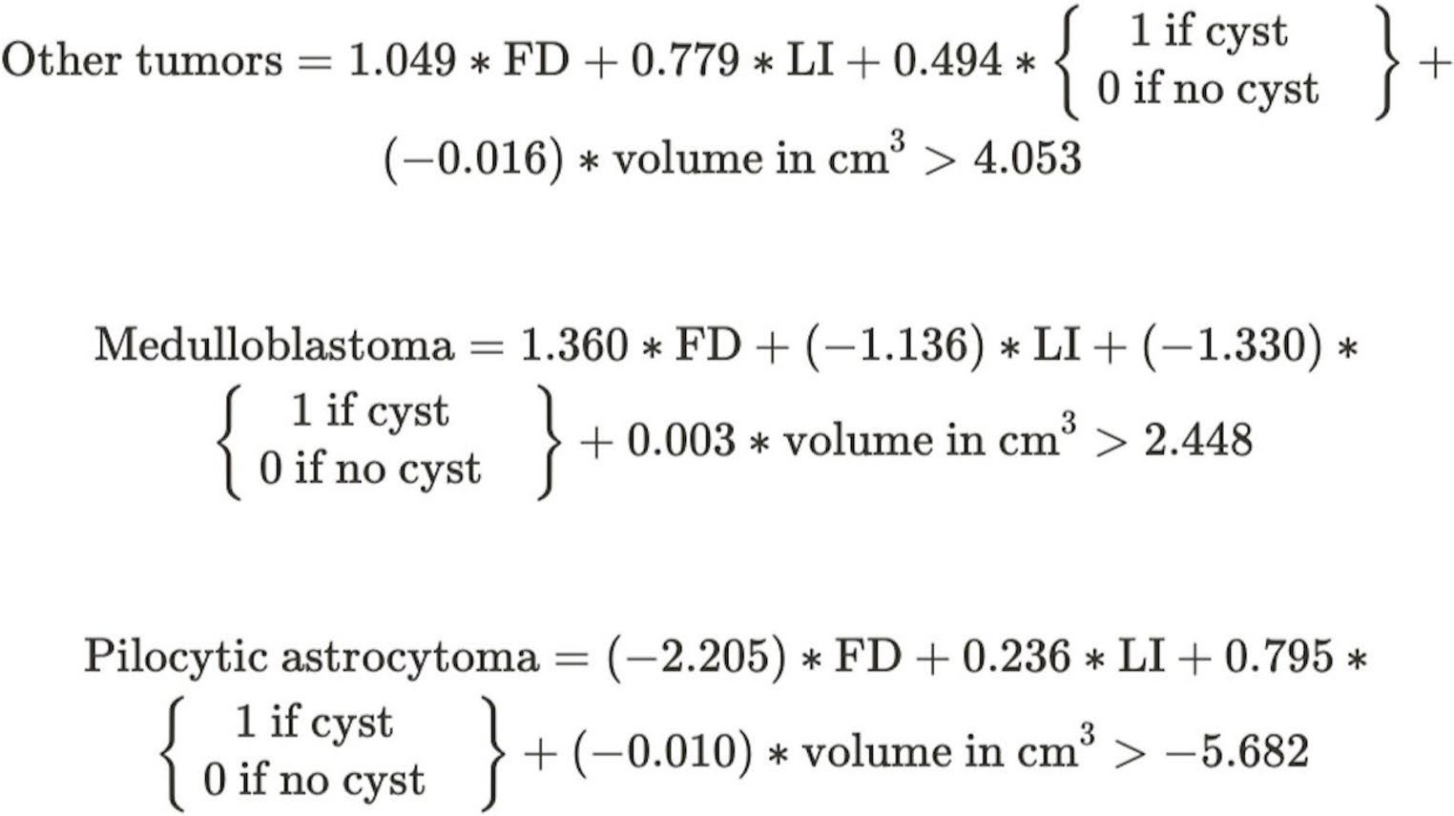
Our histopathology estimator formula (calculating all three equations for one tumor is necessary)

**Fig. 5:**
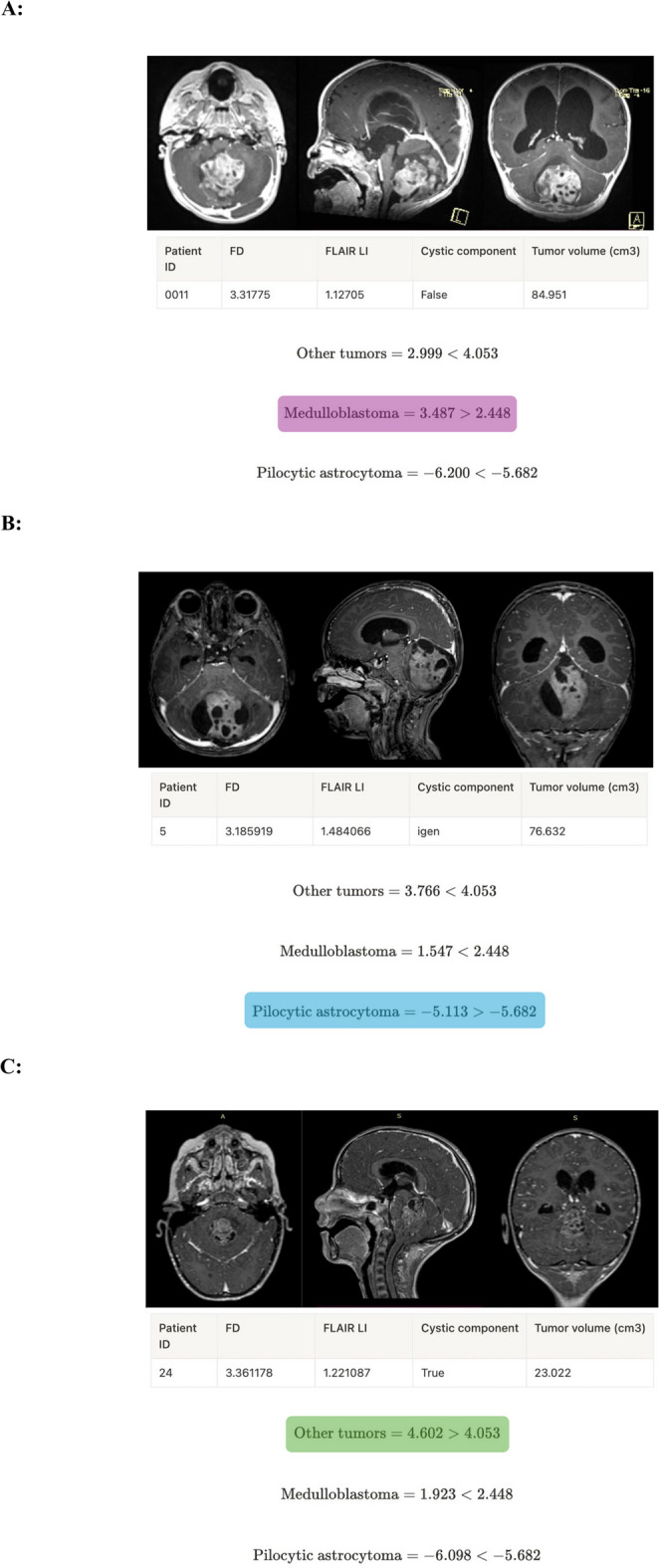
Demonstration of the use of our prediction model in three cases: **A** in a patient with medulloblastoma (T1-weighted contrast-enhanced axial, sagittal and coronal (left to right) MRI images). **B** in a patient with pilocytic astrocytoma (T1-weighted contrast-enhanced axial, sagittal and coronal (left to right) MRI images). **C** in a patient with “other tumor"” (posterior fossa ependymoma) (T1-weighted contrast-enhanced axial, sagittal and coronal (left to right) MRI images)

The prediction ability of each factor was weighted by the regression coefficients of the logistic regression analysis (Table 1), as we have three classes (“other tumor”, “medulloblastoma”, and “pilocytic astrocytoma”) with three total scores, one for each class. We created a formula for each histology subtype (Fig. 4). To calculate the total score for a given class, multiply the weights by the parameter value, and then sum them. If it is higher than the threshold for that class, the sample belongs to that class. If more than one class has a score higher than the threshold, then the one with the largest difference (‘delta’) from the threshold should be selected. If all three total scores are lower than the corresponding class thresholds, the model cannot predict the class. The exact calculation method is illustrated in Fig. 5, where examples are provided for all three tumor subtypes.

**Table 1:** Weights of the estimator formula (derived from the regression coefficient of the logistic regression analysis)

Class	Variable	Weight	OR (95% CI)	*p*-value
Other histology	FD	1.049	2.855 (0.873–10.719)	0.118
	FLAIR LI	0.779	2.179 (1.115–3.935)	0.023
	Cystic component	0.494	1.639 (0.718–4.665)	0.284
	Tumor volume (cm^3^)	-0.016	0.984 (0.980–0.990)	‹0.001
	Threshold	4.053	undefined	undefined
Medulloblastoma	FD	1.360	3.896 (1.318–11.953)	0.029
	FLAIR LI	-1.136	0.321 (0.205–0.662)	‹0.001
	Cystic component	-1.330	0.264 (0.113–0.878)	0.013
	Tumor volume (cm^3^)	0.003	1.003 (0.995–1.01)	0.468
	Threshold	2.448	undefined	undefined
Pilocytic astrocytoma	FD	-2.205	0.110 (0.038–0.424)	0.001
	FLAIR LI	0.236	1.266 (0.691–2.843)	0.504
	Cystic component	0.795	2.214 (0.949–5.323)	0.102
	Tumor volume (cm^3^)	0.010	1.010 (1.005–1.017)	0.002
	Threshold	-5.682	undefined	undefined

## Discussion

Regardless of the histological subtype, the first-line treatment option for pediatric posterior fossa tumors is maximally safe surgical resection, which ensures the best neurological function [20]. However, surgery is not without risk, various studies estimate the complication rate to be between 30% and 75% [21–23]. In the immediate postoperative period, the development and/or worsening of ataxia, gait disturbances, eye movement, speech and swallowing disorders, and lower cranial nerve dysfunction can be expected. In addition, cerebellar cognitive affective syndrome may affect a quarter of the patients [24, 25], causing a serious deterioration in quality of life, especially in the absence of appropriate rehabilitation [26].

Pilocytic astrocytomas could be potentially cured with gross total resection, but even with subtotal resection and targeted therapy, a 1-year PFS of over 85% can be expected [27]. For ependymomas, subtotal resection significantly reduces life expectancy [28]. For these two tumor types, the surgical goal is the aim of gross total resection. For medulloblastomas, maximum safe resection remains the standard of care, emphasizing that near-total resection does not negatively affect survival rates [6]. On the other hand, diffuse intrinsic pontine gliomas (DIPG) or diffuse midline gliomas (DMG) are not amenable to surgical resection due to their location in highly eloquent brainstem. Therefore, obtaining biopsy to get the exact molecular profile is essential for identifying potential targets for pharmacological therapy [29].

For pediatric posterior fossa tumors, MRI is the gold standard for accurate assessment of localization and anatomical relationships, as well as for planning surgery and estimating expected complications. However, even with the use of advanced techniques, such as diffusion, perfusion and spectroscopy, MRI still lacks the specificity to differentiate histological subgroups.

Another possible non-invasive method of preoperative diagnosis is radiomics, an emerging method that uses machine learning techniques to extract different quantitative metrics from medical images, such as lesion size, shape, texture, signal intensity, and relaxivity values. Zhang et al. [30] described the ability to select high-risk ependymoma cases based on the characteristics of T1 and T2 sequences, and the same study group [31] was able to select four relevant molecular subgroups of medulloblastoma patients using a machine learning decision pathway for radiogenomic evaluation.

Another type of information is the geometric data obtained from fractal analysis. Unlike conventional radiomics, which typically relies on fixed-scale texture features or local intensity statistics, fractal geometry offers a multi-scale assessment of tumor complexity. Fractal dimension reflects how irregular structures occupy three-dimensional space, while lacunarity captures the spatial distribution of gaps and heterogeneity within the tumor volume [7, 8]. This global, scale-invariant characterization may be especially valuable in pediatric brain tumors, where structural complexity is often linked to biological features such as cellular architecture, vascularity, and growth behavior.

Friconnet et al. [32] described an AUC value between 0.71 and 0.72 for the correlation between fractal analysis results, the WHO grade, and the brain invasion characteristics of intracranial meningiomas. Won et al. [33] were able to non-invasively predict the molecular genetic profile (TERT promoter mutation) of tumors with 84% probability (95% CI = 0.71–0.93) based on FD and lacunarity values of WHO grade 2 meningiomas.

In their study, Liu et al. [14] investigated one of the hallmark features of glioblastomas—the necrotic region—using fractal geometry-based analysis. They found that lower fractal dimension (FD) and higher lacunarity index (LI) values were significantly associated with both longer progression-free survival and overall survival. The relationship between fractal parameters derived from the necrotic region and patient prognosis was also explored by Curtin et al. [34]. Their findings demonstrated a significant correlation between the LI of the necrotic area and progression-free survival.

Liu et al. [13] were able to distinguish PCNSL from glioblastoma based on FD and lacunarity with an AUC of 0.895. Di Ieva et al. [35] performed fractal analysis on MRI susceptibility-weighted imaging scans and found a significant difference in fractal dimension values between WHO grade 4 gliomas and PCNSL.

In our single-center study, we aimed to differentiate pediatric posterior fossa tumors based on fractal characteristics, for which no previous communication was found in the literature. In addition, we created a predictive tool based on our findings (Fig. 4). Relying solely on the results of fractal analysis, we were able to differentiate the three main pediatric posterior fossa tumors based on FD and FLAIR LI; 0.716 AUC (95% CI = 0.607–0.862) was found. The predictive model was improved to 0.793 AUC (95% CI = 0.628–0.921) by including some clinical parameter (tumor volume and cystic feature of the tumor).

Small sample size and class imbalance are the main limiting factors in prediction evaluation. There is a large variance between individual bootstrap samples or cross-validation folds, and such limitations make it possible to quickly evaluate outliers that will degrade the metrics. On the other hand, hyper-optimization for a specific random split leads to overfitting, which greatly overestimates performance.

Fractal measurement with subsampling takes roughly 20 min per patient (computer properties: 2x Intel(R) Xeon(R) Platinum 8268 CPU @ 2.90 GHz, 900 GB RAM), which is a crucial limiting factor. Without subsampling, accurate measurement would take roughly two weeks using our given hardware. This slowness is mainly due to the “curse of dimensionality”. For higher dimensional objects, the number of boxes to be evaluated increases polynomially, which means that a polinomially larger number of CPU cores would be needed to keep up with the resource demands.

To sum up, we have shown that fractal dimension and lacunarity measurements are indicative predictors of non-invasive information for histopathology, and when combined with tabular structural imaging data, the multimodal feature set is a helpful decision support tool.

### Limitations

Our study has several important limitations that may affect the reliability and generalizability of fractal measurements:

- Retrospective nature of the study.
- Small sample size of 44 patients with varied pathologies, leading to a class imbalance that may affect model generalizability.
- Case classification was based on the histopathological diagnosis extracted from the layered report.
- Segmentation-related uncertainties: Manual tumor segmentation, despite multi-reader validation (κ = 0.899), introduces operator-dependent variability that may propagate through fractal calculations. Subtle differences in margin delineation, particularly in areas of peritumoral edema or enhancement, could significantly impact boundary-sensitive fractal measurements. Future studies should incorporate automated segmentation algorithms with quantitative validation metrics.
- Multi-institutional acquisition heterogeneity: Images were acquired across multiple institutions using different scanner models and protocols, with resolutions varying from 0.3 mm³ to 1 mm³ per voxel. While standardized preprocessing (resampling to 1 mm³, coregistration) was performed, this process introduces interpolation artifacts that may alter fine-scale geometric features captured by fractal analysis. The mapping between different native resolutions and our standardized grid may create artificial texture patterns, particularly affecting lacunarity measurements.
- Sequence-dependent margin definition: Tumor boundaries appear differently across MRI sequences (T1, ceT1, T2, FLAIR), and our choice of ceT1 as the registration target may not optimally capture the true tumor extent for all cases. Different sequences highlight different tissue characteristics, potentially leading to systematic variations in fractal measurements depending on sequence selection.
- Scale-limited fractal analysis: The available scales for fractal analysis are constrained by MRI resolution, resulting in uncertainty in measured fractal dimension and lacunarity values. This limitation is distinct from statistical sampling issues and affects the fundamental validity of fractal measurements. Without subsampling, calculation time is prohibitive (1 week per patient); with subsampling (1:100), measurement precision may be compromised.

## Conclusion

Radiomics is an emerging method that provides additional information by extracting different quantitative metrics from medical images such as size, shape, texture, signal intensity and fractal features of the lesion.

Fractal analysis offers a means of uncovering previously hidden information within MRI scans, particularly in the context of pediatric posterior fossa tumors. Fractal geometry offers a powerful tool to characterize the intricacies of irregular and rough shapes inherent in these tumors. Fractal dimension serves as a concise metric, encapsulating the complexity of the shape of an object. It thus allows for meaningful comparisons between different patient cohorts. To the best of our knowledge, this is the first time that we report a scoring system based on a combination of fractal measures and clinical parameters to aid in preoperative preparations.

Data from 44 children showed that the 3 main tumor types (pilocytic astrocytoma, medulloblastoma, and other tumors) were significantly distinct in terms of fractal dimension and FLAIR lacunarity property. A scoring system was constructed by merging fractal measures with conventional imaging parameters, which may assist in improving preoperative diagnostic considerations. However, given the relatively small sample size, these findings should be interpreted with caution. Further studies with larger cohorts are required to validate the scoring system and determine its potential utility in clinical decision-making and patient care.

Table 1 Weights of the estimator formula (derived from the regression coefficient of the logistic regression analysis).

## Supplementary Information

Below is the link to the electronic supplementary material.

Supplementary Material 1

Supplementary Material 2

## Data Availability

The datasets used and/or analyzed during the current study are available from the corresponding author upon reasonable request.

## Code Availability

FracND - https://gist.github.com/baskayj/0df1ba668307d9fe5017b8a22e7a655d.

## Abbreviations

ASL: Arterial spin labeled
AT/RT: Atypical teratoid/rhabdoid tumor
AUC: Area under the ROC curve
ce: Contrast enhancement
CI: Confidence interval
CPU: Central processing unit
DCE: Dynamic contrast enhanced
DIPG: Diffuse intrinsic pontine glioma
DMG: Diffuse midline glioma
DSC: Dynamic susceptibility contrast
FD: Fractal dimension
FLAIR: Fluid-attenuated inversion recovery
ICC: Intraclass correlation coefficient
LI: Lacunarity index
MRI: Magnetic resonance imaging
MRS: Magnetic resonance spectroscopy
OS: Overall survival
p: Probability
PCNSL: Primary central nervous system lymphoma
PFS: Progression free survival
rCBV: Relative cerebral blood volume
ROC: Receiver operating characteristic
ROI: Regions of interest
SHH: Sonic hedgehog
WHO: World Health Organization
