# Supplementary material for: Fractal-geometry analysis of pediatric posterior fossa tumors – a preoperative tool for prediction of histopathology
Source: Neurosurg Rev. 2026 Jan 22;49(1):141. doi: 10.1007/s10143-025-04078-9 (PMC12827295; doi:10.1007/s10143-025-04078-9)
Supplement: Supplementary file 1 — Supplementary Material 1 [file 10143_2025_4078_MOESM1_ESM.docx]

# 1. Distribution of scores among the bootstrap samples (n = 100)


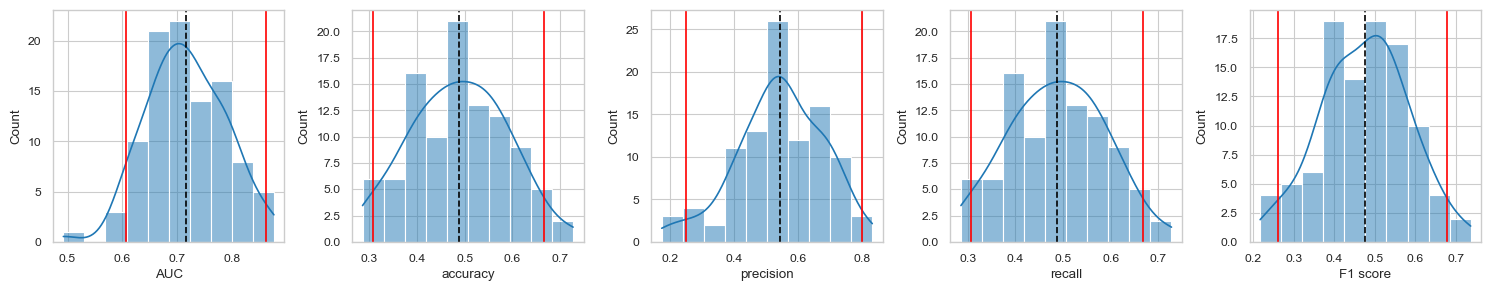


**Figure 1.:** Distribution of model metrics calculated on the out-of-boostrap samples of the logistic regression using only FD and FLAIR LI to predict histopathology. Solid blue line indicates the KDE of the distribution, red lines mark the 95% CI, dashed black line the mean.


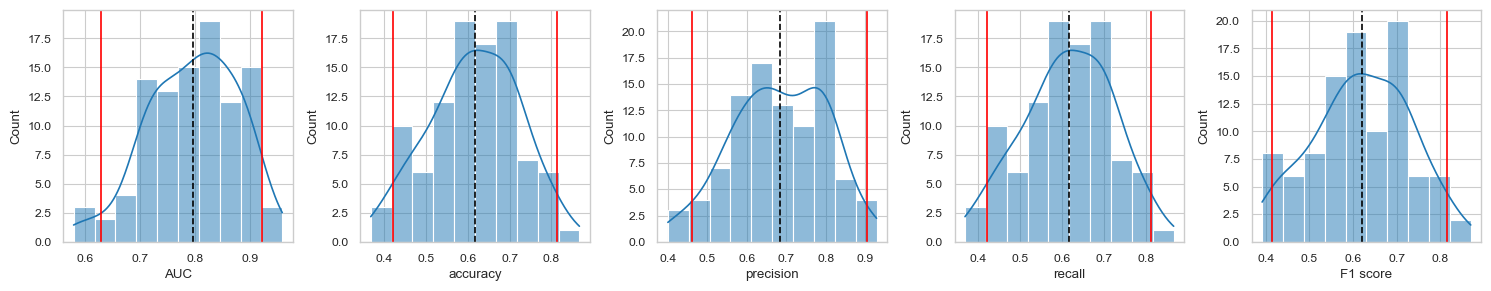


**Figure 2.:** Distribution of model metrics calculated on the out-of-boostrap samples of the logistic regression using the fractal parameters FD, FLAIR LI and clinical parameters cystic component and tumor volume to predict histopathology. Solid blue line indicates the KDE of the distribution, red lines mark the 95% CI, dashed black line the mean.
