# Supplementary material for: Fractal-geometry analysis of pediatric posterior fossa tumors – a preoperative tool for prediction of histopathology
Source: Neurosurg Rev. 2026 Jan 22;49(1):141. doi: 10.1007/s10143-025-04078-9 (PMC12827295; doi:10.1007/s10143-025-04078-9)
Supplement: Supplementary file 2 — Supplementary Material 2 [file 10143_2025_4078_MOESM2_ESM.docx]

# Histology Prediction

These evaluations used the same bootstrapping (n=100) and out-of-boot test sets as the ROC curves and metrics presented in the main manuscript.

**Calibration polts:**
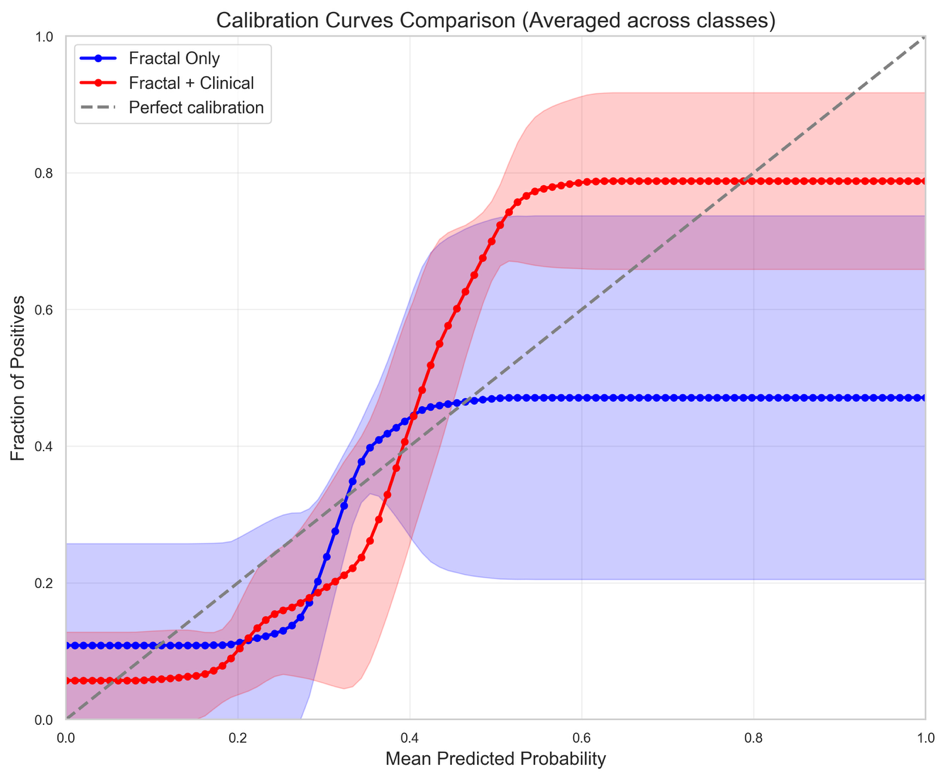

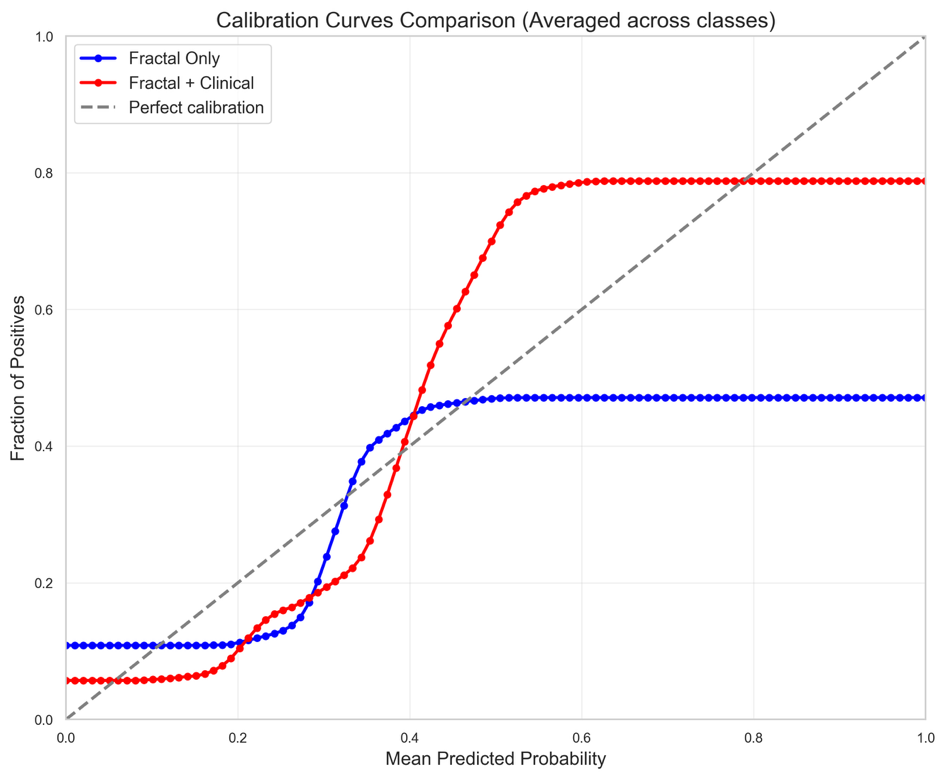


The calibration curves show the relationship between predicted probabilities and actual outcomes. This figure displays calibration averaged across all classes, showing that both models exhibit some deviation from perfect calibration. The "Fractal + Clinical" model demonstrates somewhat better calibration compared to the "Fractal Only" model, with the combined model's predictions more closely aligned with actual outcomes across different probability range, albeit with some overconfidence in the high probability ranges.

**Confusion Matrices**

There are many ways to create a confusion matrix, that summarizes multiple confusion matrices from out-of-boot samples coming from our bootstrapping methodology. We believe an honest representation can be obtained by the following:

1. Each out-of-boot confusion matrix is normalized to one row-wise. This will alleviate the varying number of classes in the out-of-boot samples.
2. We take an element-wise median. Note, that median being an element-wise statistic, the median values in the confusion matrix will no longer preserve the row-sum constraint. Though, the uncertainty intervals (meadian ± IQR/2) will always include values that satisfy the row-sum constraint.
3. We estimate the uncertainty with the inter-quantile range (IQR): *± IQR/2* (Except when the median is zero, then it’s strictly *+ IQR*

**Fractal Only**

| **True\Predicted** | **Medulloblastoma** | **Other tumors** | **Pilocytic Astrocytoma** |
| --- | --- | --- | --- |
| Medulloblastoma | 0.667±0.138 | 0.000+0.200 | 0.250±0.200 |
| Other tumors | 0.286±0.200 | 0.333±0.150 | 0.375±0.125 |
| Pilocytic Astrocytoma | 0.200±0.121 | 0.200±0.188 | 0.500±0.133 |

**Fractal + Clinical**

| **True\Predicted** | **Medulloblastoma** | **Other tumors** | **Pilocytic Astrocytoma** |
| --- | --- | --- | --- |
| Medulloblastoma | 0.667±0.150 | 0.200±0.125 | 0.000+0.250 |
| Other tumors | 0.200±0.200 | 0.500±0.140 | 0.200±0.167 |
| Pilocytic Astrocytoma | 0.143±0.125 | 0.200±0.143 | 0.667±0.093 |
